# *Helicobacter pylori* recrudescence and its influencing factors

**DOI:** 10.1111/jcmm.14682

**Published:** 2019-09-19

**Authors:** Yan Sun, Jun Zhang

**Affiliations:** ^1^ The Second Clinical Medical College Zhejiang Chinese Medical University Hangzhou China; ^2^ Department of Gastroenterology Zhejiang Provincial People's Hospital of Hangzhou Medical College Hangzhou China

**Keywords:** colonization, *Helicobacter pylori*, influencing factors, recrudescence

## Abstract

*Helicobacter pylori* (*H pylori*) is known as one of the most common infectious pathogens, with high infection and recurrence rates worldwide. The prevalence of *H pylori* is up to 90% in developing countries, while the annual recurrence rate is much higher than that in developed countries. Recurrence can occur either by recrudescence or reinfection. Compared with reinfection, the time window for recrudescence is generally shorter, followed by the recurrence of *H pylori*–associated diseases in the short‐term. Many factors are involved in the *H pylori* reinfection, such as the prevalence of *H pylori* infection, living conditions and economic development, health conditions and so forth. Previous studies focused less on *H pylori* recrudescence. Therefore, the influencing factors for *H pylori* recrudescence needed further exploration. This study reviewed the recrudescence of *H pylori* infection and its influencing factors.

## 1 INTRODUCTION

*Helicobacter pylori* (*H pylori*) is a microaerobic Gram‐negative bacterium that colonizes the human stomach and duodenum.^1^ It can cause lifelong infection without eradication. Many studies showed^2^, ^3^ that *H pylori* led to some important gastrointestinal diseases, such as chronic gastritis, peptic ulcer, gastric adenocarcinoma and mucosa‐associated lymphoid tissue lymphoma, and was associated with a variety of parenteral diseases such as idiopathic thrombocytopenic purpura. *H pylori* eradication significantly alleviated stomach inflammation, promoted ulcer healing and prevented gastric cancer.^2^ In 1994, *H pylori* was listed as Group I carcinogen. The 2015 Tokyo Global Consensus Report^4^ defines *H pylori* gastritis as an infectious disease and recommends eradication therapy for *H pylori*–infected individuals, except in the case of competing considerations. However, *H pylori* infection can still recur after eradication therapy. Recurrence can occur by either recrudescence or reinfection.^5^ Compared with reinfection, the time window for recrudescence is generally shorter. Recrudescence is generally considered as *H pylori* recurrence within 1 year after eradication, followed by the recurrence of *H pylori*–associated diseases in the short‐term.^6^, ^7^ Patients with short‐term recurrence suffer from the risk of recurrence of these diseases. The economic pressure, psychological burden and potential adverse drug reactions have increased dramatically. Therefore, exploring the factors related to *H pylori* recrudescence is important. In this study, the recrudescence of *H pylori* and its influencing factors were discussed.

## 2 DEFINITION AND DIAGNOSIS OF *H PYLORI* RECRUDESCENCE

*Helicobacter pylori* recurrence is generally divided into recrudescence and reinfection.^5^ Recrudescence is defined as the reappearance of the original infection following an initially false‐negative post‐eradication test result.^2^ A small amount of *H pylori* that has not been eradicated (*H pylori* was hidden in the deep part of the stomach or the gastric epithelial metaplasia of the duodenum) or is in dormancy (eg *H pylori* coccoid forms) is recolonized, reproduced and eventually detected. Therefore, the recrudescence strain is generally the original infectious strain. Reinfection is defined as infection with a new strain or a strain homologous to the original strain of *H pylori*.

So far, relevant studies have shown that recrudescence is generally considered as *H pylori* recurrence within 1 year after eradication.^6^, ^7^ Gisbert et al^8^ suggested that the cumulative annual recurrence rate after *H pylori* eradication was 5.3% after 1 year, 6.8% after 2 years, 7% after 3 years, 7.6% after 4 years and 9.3% after 5 years. The present study found that recurrence decreased with time and declined sharply after the first year. Kim et al^9^ performed a 2‐year follow‐up of patients with *H pylori* eradication. They found that, regardless of first‐line or second‐line therapy, the recurrence rate in the first year was significantly different (9.3% vs 4.5%) and the recurrence rate in the second year was similar (2.0% vs 2.9%). If the recurrence after *H pylori* eradication is reinfection, the annual reinfection rate should be stable.^8^, ^9^ However, the annual recurrence rate of *H pylori* increases at a steady rate, which is contrary to the conclusions of the aforementioned studies. It is generally believed that the recurrence of *H pylori* in the first year after eradication is mainly based on recrudescence. However, Raymond et al^10^ performed a strain typing study on three patients with repeated recurrence after *H pylori* eradication. The time interval between the first recrudescence of two patients was 2 years, and the recrudescence interval was 1‐3 years. One patient's first reinfection interval was up to 8 years and the reinfection interval was 1‐2 years. Accordingly, Raymond et al believed that the recurrence interval was not a reliable clinical marker for recrudescence. However, the number of specimens in the aforementioned study was extremely small, with some contingency in the conclusion. Further large‐sample *H pylori* strain typing follow‐up studies are needed to confirm this view.

To distinguish whether *H pylori* recurrence is recrudescence or reinfection, genotyping methods are used to judge the *H pylori* strain type before and after recurrence. *H pylori* genotyping methods include^11^ multi‐locus sequence typing (MLST), pulsed‐field gel electrophoresis (PFGE), random amplification of polymorphic DNA (RAPD), amplified fragment length polymorphism (AFLP), whole‐genome sequencing (WGS) and so on. Multi‐locus sequence typing analyses strain variation by polymerase chain reaction (PCR) amplification of multiple housekeeping genes (such as atpA, efp, mutY and so on) and determination of their nucleic acid sequences.^12^ Multi‐locus sequence typing has the advantages of high repeatability and high resolution and can provide more detailed information on human migration than human genetic analysis to a certain extent.^12^ However, MLST only reflects the variability of several housekeeping genes. Pulsed‐field gel electrophoresis is to detect some large fragments of linear DNA and is considered as the gold standard for bacterial typing.^13^ But it has not been widely used in *H pylori* typing. It is very crucial of restriction enzymes choice and enzyme digestion condition control, which still needs further exploration in exploration in *H pylori* typing.^13^ Random amplification of polymorphic DNA is a typing technique based on PCR that can perform polymorphism analysis on the entire unknown sequence genome. Even trace amounts of DNA can also be analysed. But there still exist some limitations. Random amplification of polymorphic DNA cannot provide any information about strain virulence factors and genetic evolution information.^14^ Meanwhile, it depends highly on the quality and quantity of the template.^14^ Amplified fragment length polymorphism is a molecular marker technology developed on the basis of PCR, which has the advantages of high repeatability and high resolution.^15^ However, it also has high‐quality requirements for DNA template and it is a non‐rapid detection method.^15^ Whole‐genome sequencing masters the entire genomic sequence of the microorganism. In theory, any microorganism can be typed with a resolution of a single base.^13^ However, at the same time, the experiment cost is large and the cycle is long. Therefore, the aforementioned gene detection methods have not been widely carried out clinically. In addition, some studies pointed out that even if the same *H pylori* strain was identified before and after recurrence, still the patient might be reinfected by the same strain in the environment.^16^ Therefore, how to quickly and effectively identify *H pylori* recurrence and recrudescence is a hot issue worthy of further study.

## 3 INFLUENCING FACTORS OF *H PYLORI* RECRUDESCENCE

The rates varied widely among countries and areas from a high of 21.3% to a low of 0.2%.^5^ In recent years, more attention has been paid to *H pylori* and its diseases at home and abroad. However, Hu et al^17^ performed a systematic review with meta‐analysis of *H pylori* recurrence rates worldwide. They suggested no change in the recurrence rates over the past 27 years.^17^ These findings showed that the studies on the prevention and treatment of *H pylori* recurrence might not have achieved great results.

**Figure 1:**
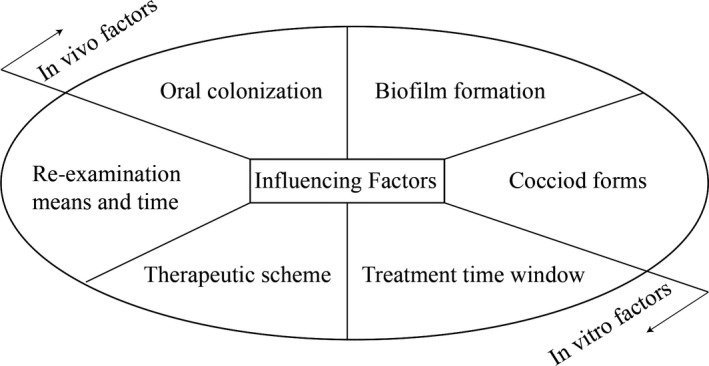
Influencing factors of *Helicobacter pylori* recrudescence. The factors involved in *H pylori* recrudescence can be roughly divided into two categories: (1) false‐negative results of the review (mainly in *vivo* factors); and (2) small amounts of unkilled *H pylori* or dormant *H pylori* lurking in the human body (mainly in *vitro* factors). *H pylori* itself is a major component of the in *vivo* factors including its oral colonization, biofilm formation and coccoid forms transformation. Re‐examination means and time, therapeutic scheme and treatment time window as in *vitro* intervention are also involved in *H pylori* recrudescence. Both in *vivo* and in *vitro* factors result in *H pylori* recrudescence

The global annual recurrence rate of *H pylori* was 4.3%.^17^ The annual recurrence rate of *H pylori* in developing countries (13%) is much higher than that in developed countries (2.7%).^18^ Among studies on *H pylori* recurrence factors, reinfection studies are relatively mature. It is generally believed that reinfection mainly involves host factors such as total *H pylori* infection rate, personal hygiene habits and so on. The factors involved in *H pylori* recrudescence can be roughly divided into two categories (Figure 1)^16^: (1) false‐negative results of the review (mainly in *vivo* factors); and (2) small amounts of unkilled *H pylori* or dormant *H pylori* lurking in the human body (mainly in *vitro* factors). The influencing factors for *H pylori* recrudescence will be discussed next.

### 3.1 Selection of therapeutic scheme and treatment time window

Many studies have reported that the therapeutic scheme is closely related to *H pylori* recurrence within 1 year.^8^, ^9^ A therapeutic scheme with low *H pylori*‐eradication rate is temporary clearance rather than complete eradication, leading to *H pylori* recrudescence. Therefore, the rate of *H pylori* recrudescence negatively correlates with the eradication rate. The lower the *H pylori*‐eradication rate, the higher the *H pylori* recrudescence rate. Gisbert et al^8^ performed a prospective study involving 1000 patients, selecting two therapeutic schemes with low eradication rates (omeprazole plus amoxicillin, 32%; omeprazole plus amoxicillin and metronidazole, 56%) and two therapeutic schemes with high eradication rates (omeprazole plus clarithromycin and either amoxicillin or metronidazole, 85%; bismuth subcitrate, tetracycline chlorhydrate and metronidazole, 77%). The review results after 1 year showed that the former *H pylori* recrudescence rate was significantly higher than the latter (11.3% vs 4.7%, *P* = .006). A similar study was conducted in Korea, in which a standard triple therapy (eradication rate was 79.9%) and a barium‐containing quadruple therapy (eradication rate was 90.4%) were used for *H pylori* eradication.^9^ A follow‐up showed that *H pylori* recrudescence rates were 9.3% and 4.5% (*P* < .05) within 1 year.^9^ However, due to the geographical variation in *H pylori* resistance to antibiotics, even in the same treatment programme, *H pylori*‐eradication rates were different in different areas and countries.^19^, ^20^ Therefore, to increase the *H pylori* eradication and reduce its recurrence, different countries should use drugs as a first‐line treatment based on the epidemiological study of local *H pylori* antibiotic resistance.

In addition to therapeutic schemes, selecting an appropriate treatment time window is also important for the *H pylori* eradication. A meta‐analysis of China in 2017 (43 studies, 7686 patients) suggested that the 10‐day or 14‐day treatment of sputum quadruple therapy significantly improved *H pylori* eradication compared with its 7‐day therapy.^21^ Prolonging bismuth‐containing quadruple therapy from 10 to 14 days did not show better efficacy. Yuan et al^22^ analysed 59 studies based on a proton pump inhibitor (PPI) triple therapy and concluded that, regardless of the antibiotics type and its dose, an increase in the administration time of PPI triple therapy from 7 to 14 days significantly increased *H pylori*‐eradication rate (72.9% vs 81.9%, *P* < .05). Although the eradication rate increases with a prolonged administration time, the subsequent adverse drug reactions may also increase. Therefore, which *H pylori* therapeutic scheme to choose and how to choose the appropriate treatment time window to minimize *H pylori* recrudescence still need exploration. The direct relationship between the administration time for *H pylori*‐eradication therapy and *H pylori* recrudescence requires further clarification.

### 3.2 Re‐examination means and time

The detection methods for *H pylori* are of two types: invasive and non‐invasive. Invasive detection methods include rapid urease test, HE staining, Giemsa stain, bacterial culture and so on. The non‐invasive detection methods include a 13C‐urea breath test, 14C‐urea breath test, stool antigen test and so on. However, if only one of the aforementioned means is used to evaluate the efficacy, sensitivity and specificity are reduced. If a patient has used a PPI 2 months before re‐examination or an antibiotic 1 month before re‐examination, the breath test may be false negative because of the urease activity suppressed by these drugs. Especially, when the result is at a critical value, whether to use eradication therapy is difficult to determine.^20^ In addition, due to the drugs such as PPI, the *H pylori* distribution in the stomach changes (eg *H pylori* in the antrum is moved up to the corpus ventriculi). *H pylori* rejuvenates and multiplies due to the reduction or loss of drug efficacy after a period of eradication therapy. When *H pylori* is detected again, the result becomes positive again, which is *H pylori* recrudescence. Therefore, to reduce diagnostic error, it is best to use two or more different diagnostic techniques for *H pylori* recurrence detection.

In addition to *H pylori* detection methods, the evaluation time for *H pylori* eradication is also related to *H pylori* recrudescence. At present, the clinical evaluation of *H pylori* eradication is carried out at least 4 weeks after the therapy completion. Neil et al^23^ also believed that 1 month after eradication therapy was sufficient to evaluate the effect of *H pylori* eradication. Some studies^16^, ^24^ postponed the re‐examination means to 2 months to reduce the false‐negative rate. However, *H pylori* can be recrudesced within a few months after therapy. Ishizuka et al^25^ found that *H pylori* could reappear within 3 months after eradication therapy. When a patient is examined within 2‐3 months after eradication, it is difficult for the investigators to distinguish between eradication failure and recrudescence. Therefore, the selection of a time node for re‐examination influences the evaluation of *H pylori* recrudescence. However, a few studies currently define an evaluation period for *H pylori* eradication failure and recrudescence.

### 3.3 *Helicobacter pylori* oral colonization

*Helicobacter pylori* is mainly transmitted through multiple routes between humans, including faecal‐oral transmission, oral‐oral transmission, gastric‐oral transmission and iatrogenic transmission. It can also be transmitted to humans through water, environment and animals.^26^, ^27^ Therefore, *H pylori* in the oral cavity may play an important role in gastric *H pylori* transmission. Certain microaerobic environments are suitable for *H pylori* survival in the oral cavity, such as plaque, root canal and so on. Some studies have shown a link between oral *H pylori* and gastric *H pylori* (Table 1). Naviba et al^28^ included 23 studies (1861 patients) and found that the per cent of agreement between the dental plaque *H pylori* status and the gastric *H pylori* was estimated as 82%. Therefore, it is considered that oral *H pylori* and gastric *H pylori* have homology. Further, *H pylori* oral colonization may be one of the risk factors for gastric *H pylori* recrudescence. In addition, a meta‐analysis in 2011^29^ showed that the prevalence of *H pylori* infection in the oral cavity in gastric *H pylori*–positive patients was significantly higher than that in gastric *H pylori*–negative patients (45% vs 23.9% OR 3.61, *P* < .0001). The eradication efficiency in stomach and oral cavity is 85.8% and 5.7%, respectively (OR 55.59, *P* < .00001). It shows that the clinical routine *H pylori* therapeutic scheme has a weak killing effect on oral *H pylori*. Bouziane et al^30^ performed a meta‐analysis (sample size: 298 cases) and evaluated the effect of periodontal therapy on the prevention of gastric *H pylori* recurrence. They found that, compared with the eradication therapy alone, the adjunction of periodontal therapy significantly reduced the relative risk of persistence of gastric *H pylori* by 63% (OR 0.37, *P* = .0004) in patients with gastric diseases. A recent prospective randomized trial in Thailand (sample size: 698 cases) also drew the same conclusion.^31^ After the eradication of gastric *H pylori* infection, the recurrence of gastric *H pylori* was significantly lower in the group receiving gastric *H pylori* treatment plus periodontal therapy than in that receiving gastric *H pylori* treatment alone (PP analysis: OR 0.69, *P* = .001; ITT analysis: OR 0.67, *P* = .001), while the eradication rates were not significantly different (PP analysis: OR 0.77, *P* = .078; ITT analysis: OR 0.87, *P* = .076). Therefore, oral *H pylori* may be one of the factors for *H pylori* recrudescence in the stomach.

**Table 1:** The potential correlation between oral *Helicobacter pylori* and gastric *H pylori*

Author	Type of study	sample size	Direction of study	Methods	Country	Index	Rate	*P* value	Summary of conclusion
Assumpcao et al^44^	Cross‐sectional study	99	Genotype	RUT, PCR	Northern Brazil	Gene agreement rate	89.0%	—	Significant association between oral *H pylori* and gastric *H pylori*
Ogunbodede et al^45^	Cross‐sectional study	66	Colonization	culture, histological examination	Nigeria	Colonization correlation	—	.01	The correlation (Spearman's) between gastric and oral *H pylori* colonization was significant
Roman‐Roman et al^46^	Cross‐sectional study	196	Genotype	PCR, histological examination	Mexico	Gene agreement rate	51.1%	—	*H pylori* might reach the stomach from oral cavity
Abadi et al^47^	Cross‐sectional study	132	Colonization	PCR, culture	Iran	Prevalence of *H pylori* (oral *H pylori* vs gastric *H pylori*)	100% vs 54.2%	.001	Patients who previously infected with *H pylori* and cured were still carrying oral *H pylori*
Zou et al^29^	Meta‐analysis	1088	Eradication	RUT, PCR, UBT, CLO test, histological examination	China	Eradication rate (gastric *H pylori* vs oral *H pylori*)	85.8% vs5.7%	<.00001	Oral *H pylori* was difficulty to eradication
Jia et al^48^	Cohort study	110	Colonization	UBT	China	Prevalence of gastric *H pylori* (oral treatment vs no oral treatment)	19.5% vs 84.3%	<.05	Oral treatment was associated with lower gastric recurrence by *H pylori*
Zaric et al^49^	Cohort study	98	Eradication	PCR	Serbia	Gastric *H pylori*‐eradication rate (oral treatment vs no oral treatment)	77.3% vs 47.6%	.044	Treated with the combined therapy exhibited successful eradication of gastric *H pylori*
Song et al^50^	Cohort study	431	Eradication	UBT, HPS	China	Gastric *H pylori*‐eradication rate (oral treatment vs no oral treatment)	94.7% vs 78.4%	.012	Oral treatment might improve the eradication rate of gastric *H pylori*
Liu et al^51^	Case‐control study	443	Colonization	RUT, PCR, histological examination	China	Prevalence of gastric *H pylori* (oral *H pylori* positive vs negative)	80.1% vs 46.6%	<.01	Oral *H pylori* showed concomitant stomach infection
Rasmussen et al^52^	Cross‐sectional study	78	Colonization	Southern blotting	Brasil	Prevalence of oral *H pylori* (gastric *H pylori* positive vs negative)	71.2% vs 50.0%	<.0001	Oral *H pylori* showed a potential association with gastric reinfection
Anand et al^53^	Case‐control study	134	Colonization	RUT, HPS, histological examination	India	Prevalence of gastric *H pylori* (oral *H pylori* positive vs negative)	89.2% vs 71%	<.05	*H pylori* in oral cavity was seldom eliminated by *H pylori*‐eradication therapy

Abbreviations: CLO test, Campylobacter‐like organism test; HPS,* H pylori* antigen test; PCR, polymerase chain reaction; RUT, rapid urease test; UBT, urea breath test.

However, the conclusion may not be applicable to all populations because the studies involved a small sample size, some were limited to people of certain areas, and some involved different material parts of obtaining oral *H pylori* (such as dental plaque and saliva). Therefore, multi‐centre and large‐sample studies are needed to confirm the effect of oral *H pylori* treatment on reducing gastric *H pylori* recrudescence in the future.

### 3.4 *H pylori* coccoid forms

All living things have their own unique survival mechanisms in harsh environments, and *H pylori* is no exception. Morphologically, three forms of *H pylori* are presently considered to exist: spiral form, intermediate V‐ and U‐forms and coccoid form. Among them, the coccoid form is divided into two subtypes. Type A has irregular edges with a rough surface and is considered to be a dead form, while type B has a smoother surface, is smaller and is considered to be a dormant form.^2^, ^32^ A previous study has confirmed^33^ that coccoid *H pylori* exists in the stomach and duodenum, and the number in the duodenum is higher than that in the stomach. It indicated that the coccoid *H pylori* presence is related to the environment. When *H pylori* is exposed to harsh environments (use of antimicrobial agents, pH of the living environment, changes in oxygen content and so on), spiral *H pylori* can be converted into coccoid *H pylori*, which is the so‐called dormant form. Also, coccoid *H pylori* continues to maintain lower levels of metabolic activity, such as synthesis of urease and proteins, expression of virulence genes, and so on.^34^ However, coccoid *H pylori* cannot be recognized using traditional detection techniques or cultured and propagated in vitro. However, when the environment changes, it may be converted into the viable, culturable bacillary form that is spiral *H pylori*, and colonize and multiply in the stomach, resulting in *H pylori* recrudescence.^35^, ^36^ Coccoid *H pylori* were inoculated intragastrically in BALB/c mice. Cellini et al^37^ found that viable *H pylori* was isolated from the gastric mucosa after 2 weeks. She et al performed a similar study. When the gastric tissue mucosa was observed under an electron microscope 21 and 28 days after the last inoculation, spiral *H pylori* was found and an inflammatory reaction was observed in the pathology of stomach tissue. The aforementioned results confirmed that coccoid *H pylori* had the potential to transform into spiral *H pylori* and was the ‘seed’ of *H pylori* recrudescence.

A recent study found that coccoid *H pylori* failed to induce *H pylori* infection in mice. Boehnke et al^38^ allowed mice to freely drink water containing coccoid *H pylori* (10^9^ cells/L) and found that either prolonged exposure of the mice to drinking water or shortened euthanasia did not result in *H pylori* colonization in the mouse stomach. Further studies conducted by the aforementioned investigators revealed that even if the mice were orally administered a high concentration of coccoid *H pylori* (four times the aforementioned dose) in drinking water for 2 weeks, the infection could not be induced. However, this study used the SS1 strain, which was different from the strains selected by Cellini and She (both from the *H pylori* strain in the stomach tissue of patients with ulcers). The contradictory conclusions might be related to the different transformation ability for different strains. However, no studies have been performed on the relationship between the spiral form of different *H pylori* strains and their spiral *H pylori* transformation ability.

### 3.5 *H pylori* biofilm formation

Biofilms can be defined as adherent aggregates of microorganisms encased with an extracellular polymeric substance. Growing evidence indicates that *H pylori* can also establish biofilms.^39^, ^40^ Moreover, a study found that the *H pylori*‐eradication rate in the *N*‐acetylcysteine (NAC)‐treated group (which can eliminate and prevent biofilm establishment) before the traditional eradication therapy was significantly higher than that in the NAC‐free group (65% vs 20%, *P* = .005).^41^ Hamidian et al^42^ also believed that NAC combined with triple eradication (amoxicillin, clarithromycin and omeprazole) could increase the eradication rate of the therapeutic scheme on *H pylori* (NAC group: 72.9%; placebo group: 60.9%, *P* = .005). Moreover, in the NAC pre‐treatment group, the *H pylori* load (colony‐forming units per gram of gastric tissue) decreased by about 1 log in C57BL mice compared with the untreated group.^43^ Therefore, it was speculated that *H pylori* present in the gastric mucosa under the biofilm might be one of the risk factors for recrudescence. In addition, scanning electron microscopy revealed that most of the *H pylori* under the biofilm was coccoid.^44^ After the conventional eradication treatment, the residual biofilm *H pylori* cannot be found by traditional detection methods even if the *H pylori* colony load under the biofilm is large, and it may become the ‘seed’ for future *H pylori* recrudescence. However, no relevant clinical study has confirmed the role of biofilms in *H pylori* recrudescence.

## 4 CONTROL AND PREVENTION OF *H PYLORI* RECRUDESCENCE

*Helicobacter pylori* recrudescence is one of the main causes for *H pylori* recurrence within 1 year after the eradication therapy. Many factors are involved in *H pylori* recrudescence, such as therapeutic scheme, treatment time window, re‐examination time and means, oral *H pylori*, *H pylori* coccoid forms, *H pylori* biofilm and so on. Therefore, to reduce the chance of *H pylori* recrudescence, a therapeutic scheme with high eradication rate or a suitable therapeutic scheme according to local antibiotic resistance, and a variety of re‐examination means should be chosen to reduce the false‐negative rate. In the meanwhile, strengthening patient education, improving patient compliance and increasing the *H pylori*‐eradication rate are necessary to avoid the limitations of drug selection and drug resistance in the second eradication. The relationship between *H pylori* oral colonization and *H pylori* recrudescence has not yet reached a consensus. However, an increasing number of studies have confirmed that oral nursing and periodontal treatment may reduce the *H pylori* infection and recrudescence rates in the stomach. Multi‐centre, large‐sample studies are required to increase the reliability of evidence in the future. The aim should be to disrupt the protection mechanism of *H pylori* in a harsh environment, prevent its transformation into coccoid forms and biofilm formation and improve the *H pylori* detection rate under the protection mechanism.

## CONFLICT OF INTEREST

The authors declare that they have no conflicts of interest concerning this study.

## AUTHOR CONTRIBUTIONS

YS and JZ collected and read references. YS contributed to manuscript preparation. JZ revised the manuscript. All authors approved the final manuscript.
